# The Need for Integration of Religion and Spirituality into the Mental Health Care of Culturally and Linguistically Diverse Populations in Australia: A Rapid Review

**DOI:** 10.1007/s10943-023-01761-3

**Published:** 2023-02-13

**Authors:** Shikha Malviya

**Affiliations:** School of Health, Medical and Applied Sciences, Central Queensland University, Rockhampton, QLD Australia

**Keywords:** Culturally and linguistically diverse, CALD, Mental health care, Religion, Spirituality

## Abstract

Australia is a multicultural and linguistically diverse country. Despite the vital role of religion/spirituality in the mental health of people from culturally and linguistically diverse (CALD) backgrounds, it is not commonly included in their mental health care. A rapid review was conducted to critically evaluate the studies that identified relevant religious/spiritual aspects regarding mental health care for CALD communities. After a systematic search and screening, sixteen empirical studies were included. The findings of the review suggest that people from CALD backgrounds draw strength and comfort from their religion to support mental health. Religious/spiritual practices were identified as effective mental health strategies by CALD people. Religious leaders were noted to be important sources of mental health support and guidance for individuals from some CALD communities. Collaboration of religious leaders in the mental health care, and integration of religious/spiritual practices into mainstream mental health interventions, may improve mental health care for people with CALD backgrounds.

**Supplementary Information:**

The online version contains supplementary material available at 10.1007/s10943-023-01761-3.

## Introduction

Australia is one of the most culturally and linguistically diverse countries in the world (Australian Bureau of Statistics, 2022a). From 2017 to 2021, more than one million people arrived in Australia, contributing to the 51.5% of residents who were either first or second-generation born overseas (Australian Bureau of Statistics, 2022a). About 5.5 million people in Australia speak a language other than English at home and a substantial number of people do not speak English at all (Australian Bureau of Statistics, 2022b). The term ‘culturally and linguistically diverse’ (CALD) refers to people born overseas, people with limited English proficiency, and children of people born overseas (Khatri & Assefa, 2022). Besides linguistic diversity, in the last 2 decades, there has been a change in religious affiliation in the Australian population. Attributing to recent migration and humanitarian entrants in the past 25 years, the percentage of people reporting an affiliation with *other religions* (other than Christianity) has increased from 3.5% in 1996 to 10% of the population in 2021 (Australian Bureau of Statistics, 2022c). In 2021, the most common religions were: Christianity (43.9%); No religion (38.9%); Islam (3.2%); Hinduism (2.7%); and Buddhism (2.4%) (Australian Bureau of Statistics, 2022c).

People from CALD backgrounds have a higher likelihood of mental health concerns due to the trauma experienced before migration, the stressful process of adjustment to the new culture migrants and limited language proficiency (Abbott & Silles, 2016; Minas et al., 2013). Furthermore, stresses such as difficulties in understanding the new system to access essential services in the host country, isolation and disconnection from family and other social support pose additional challenges for people from CALD backgrounds (Bhugra, 2004; Khawaja, 2007). Though there are no clear data on the prevalence of mental disorders (Said et al., 2021), about a quarter of a million first-generation adult Australians from CALD backgrounds are estimated to experience some form of mental disorder in 12 months (Department of Health & Aged Care, 2018).

Despite the struggles of immigration and mental health challenges, people from CALD communities do not access mental health services (Au et al., 2019; Fauk et al., 2022). While there are numerous factors of nonengagement, such as shame and stigma attached to mental illness (Said et al., 2021), lack of bilingual services (Mitchell et al., 1998; Tobin, 2000) and mistrust in the services (Said et al., 2021), people from CALD background deemed mental health services not compatible to their religious and cultural beliefs (Au et al., 2019; Carolan & Cassar, 2010; Tobin, 2000).

Religion/spirituality plays an important role in the lives of people from CALD backgrounds (Bairami et al., 2021). For instance, in a comparative study, Muslim Australians from culturally and linguistically diverse (CALD) backgrounds endorsed religious beliefs more strongly than Muslims from non-CALD, Anglo/Caucasian backgrounds (Bairami et al., 2021). Religion/spirituality is related to greater psychological well-being for people from CALD backgrounds (Chan, 2009; Hashemi et al., 2020), and they often prefer religious/spiritual interventions over mainstream mental health treatments (Bairami et al., 2021; Omar et al., 2017). In a study, participants from CALD backgrounds rated psychological counselling as less effective but rated Quran recitation as significantly more effective than non-CALD participants (Bairami et al., 2021). Similarly, in a qualitative study of Muslim refugees, primarily from Somalia, participants stated that traditional "talking therapies" were ineffective and preferred religious interventions over mainstream mental health interventions (Omar et al., 2017).

In view of the important role religion/spirituality plays in the mental health of people from CALD backgrounds (Ahmad et al., 2022), religious and spiritual aspects need to be an integral part of their mental health care (Beaini & Shepherd, 2022). There is a lack of policies that address the unique mental health needs of CALD populations (Minas et al., 2013). While specialised state transcultural mental health policies provide comprehensive information regarding mental health issues of CALD populations, general Commonwealth and State and Territory mental health policies currently do not have the provision for the inclusion of religion and spirituality in the mental health care for the people from CALD backgrounds.

## The Present Study

This study aims to critically review the current evidence regarding the need for the inclusion of religion and spirituality in the mental health care of the CALD population in Australia. Since the review is about mental health care, studies relevant to mental health, including mental health resilience, and mental health outcomes were included in the review. It is anticipated that the findings of the review may highlight the importance of the inclusion of religious and spiritual aspects in mental health care for people from CALD backgrounds. The insight gained from the review may be used for the provision of evidence-based mental health care and relevant policy changes for CALD communities in Australia.

## Method

### Design and Protocol Registration

This rapid review used systematic methods to search and critically appraise published research conducted in Australia since the year 2000. Rapid reviews employ a systematic and rigorous methodological approach but may compromise the breadth or depth of the process (Khangura et al., 2012). The process of this review was guided by the World Health Organisation's practical guide on rapid reviews (Tricco et al., 2017). The protocol of the review was developed and registered with Open Science Framework (Malviya, 2022). The findings of the review were summarised using narrative synthesis to answer the research question.

### Information Sources and Search Strategy

Two databases, CINAHL and PsycINFO, were searched. A systematic and comprehensive search strategy was developed using a combination of key terms and MESH terms. The search strategy was adapted in accordance with the guidelines of individual databases. See Table 1 for an example search strategy for CINAHL. The search strategy for PsycINFO is available in Appendix 1. Searches were limited to the English language and studies published in the year 2000 or later were included in the review. Unpublished studies, case reports and grey literature were not included in the review.

**Table 1:** Search strategy of CINAHL

DATABASE	CINAHL (from 2000 to 2022)
STRATEGY	#1 AND #2 AND #3 AND #4
1	(MH “Mental Disorders +”) OR (MH “Anxiety +”) OR (MH “Depression +”) OR (MH “Stress +”) OR (MM “Psychological Well-Being”) OR (MM “Quality of Life”) OR Resilience OR Coping
2	(MH “Immigrants +”) OR (MM “Refugees”) OR “culturally diverse” OR “linguistically diverse” OR CALD OR “non-English speaking” OR migrant OR refugee OR ethnic OR "Chinese Australian" OR "Indian Australian" OR "Arabic Australian" OR "African Australian" OR "Culturally and linguistically diverse" OR “multicultural” OR “Indian” OR “Chinese” OR “Malaysian” OR “Vietnamese” OR “Arab” OR “Iraq” OR “Iran” OR “Syrian” OR “Afghan” OR “Middle Eastern” OR “Sudanese” OR “Jordan” OR “Somalian”
3	(MH "Religion and Religions +”) OR (MH "Spirituality") OR (MH "Spiritual Well-Being (Iowa NOC)") OR (MH "Spiritual Healing +”) OR (MH "Spiritual Care") OR Prayer OR Spiritual practices OR Religious practices OR Religi* OR Spirit*
4	Australia OR Australian OR Australians
Limiters applied	English language, peer-reviewed, studies conducted in Australia
Search results on 10/08/2022	66

### Study Selection and Eligibility Criteria

Studies of qualitative/quantitative/mixed experimental design that considered mental health, mental health resilience or mental health outcomes of people from CALD backgrounds were included. Only peer-reviewed studies conducted in Australia were considered. Detailed eligibility criteria are presented in Table 2. Screening of studies was conducted in two phases. The author assessed titles and abstracts against eligibility criteria in the first phase. Full-text articles were screened in the second stage.

**Table 2:** Eligibility criteria

	Inclusion	Exclusion
Population/type of participants	Adults from, culturally and linguistically diverse CALD, non-English speaking, from the minority ethnic group, migrants. Clinical or non-clinical population	Studies related to infants/children, studies related to non-CALD populations
Context	Studies that reported the role of religion/spirituality in dealing with mental health issues or identified the need for inclusion of religion/spirituality in mental health assessment and/or intervention	Studies not reporting the role of religion/spirituality in dealing with mental health issues
Outcome	Mental health, mental health resilience, mental health disorders, mental well-being	Physical conditions, any other condition not related to mental health
Study design	Observational and experimental studies—quantitative and qualitative	Opinion pieces, discussion papers, reviews, unpublished literature, textbook
Other	Peer-reviewed, in English. Studies published in 2000 or later. Studies conducted in Australia	Not peer-reviewed, a language other than English. Studies published before 2000. Studies conducted outside of Australia

### Quality Appraisal

The quality of the studies was appraised using the Mixed Method Appraisal Tool (MMAT) (Hong et al., 2018). Developed for the assessment of the methodological quality of empirical studies, the latest version of MMAT includes two screening questions and five criteria for each of the following five study designs (Hong et al., 2018). These study designs are: (a) qualitative; (b) randomised controlled trial; (c) nonrandomised; (d) quantitative descriptive; and (e) mixed methods studies (Hong et al., 2018). An overall score of the quality appraisal of each study and detailed appraisal is presented.

### Data Extraction and Analysis

Data were extracted and tabulated under the categories; (a) author/s, year, and place; (b) participants’ characteristics; (c) study design; and (d) relevant findings. Data were synthesised using narrative synthesis. Narrative synthesis is the process of formulation of an integrated interpretation of findings from multiple sources (Popay et al., 2006).

## Results

### Search Results

**Fig. 1:**
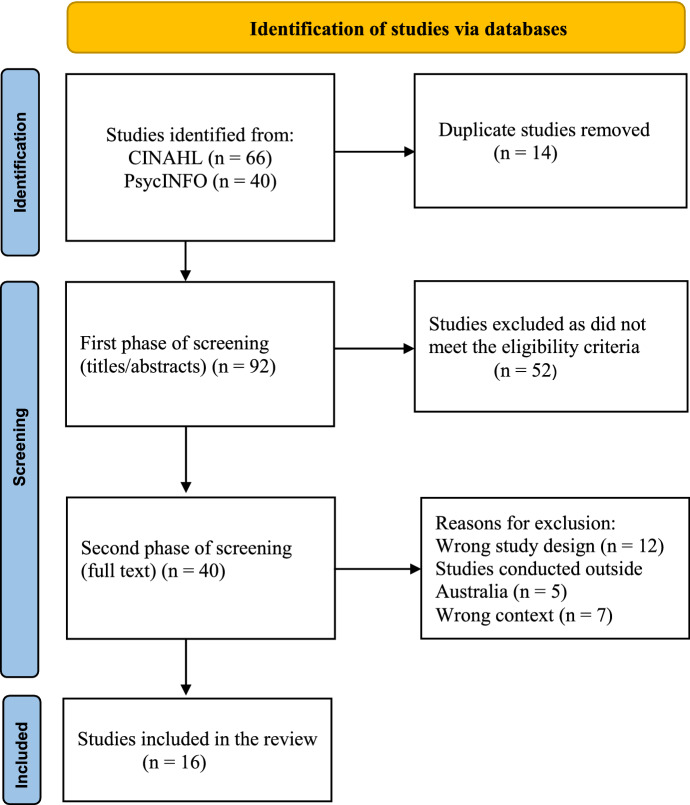
PRISMA flow diagram of screening (Page et al., 2021)

Searches of both databases retrieved a total of 106 studies. After removing 14 duplicates, 92 studies were screened in phase one, and 40 full-text studies were screened in phase two. Of these 40 studies, 24 were further excluded. Results of the searches, screening process and reasons for exclusion are outlined in Fig. 1. Sixteen studies were included in the final review.

### Result of Quality Appraisal

Results of MMAT quality appraisal of individual studies are outlined in Appendix 2. One study was identified as mixed methods (Chan, 2009); 11 as qualitative studies (Brijnath, 2015; Fauk et al., 2022; Hocking, 2021; Khawaja et al., 2008; Mitha & Adatia, 2016; Omar et al., 2017; Prasad-Ildes & Ramirez, 2006; Ridgway, 2022; Said et al., 2021; Schweitzer et al., 2007; Youssef & Deane, 2006), and four as quantitative studies (Bairami et al., 2021; du Plooy et al., 2019; Hashemi et al., 2020; Stolk et al., 2015). All included studies addressed both screening questions and stated research aims and justified findings to answer research questions. Overall, all studies met three or more of the five quality appraisal criteria of MMAT except one study (Prasad-Ildes & Ramirez, 2006). In this study (Prasad-Ildes & Ramirez, 2006), while the qualitative data collection was adequate to address the research question, there was no explanation about the approach used for qualitative data analysis.

### Characteristics of Included Studies

All of the included studies were conducted in Australia over the last 16 years in various locations. Two studies (Bairami et al., 2021; Du Plooy et al., 2019) were conducted online, while locations were not reported in the other two (Mitha & Adatia, 2016; Stolk et al., 2015). Locations of the other studies are listed in Table 3. Eleven studies used qualitative methodology (Brijnath, 2015; Fauk et al., 2022; Hocking, 2021; Khawaja et al., 2008; Mitha & Adatia, 2016; Omar et al., 2017; Prasad-Ildes & Ramirez, 2006; Ridgway, 2022; Said et al., 2021; Schweitzer et al., 2007; Youssef & Deane, 2006), four were quantitative survey studies (Bairami et al., 2021; du Plooy et al., 2019; Hashemi et al., 2020; Stolk et al., 2015), and one study used mixed method (Chan, 2009). Details of individual studies are outlined in Table 3.

**Table 3:** Characteristics of included studies

Study/year/place of study	Experimental/control or comparison (age range or mean age/sex/number of participants	Diagnosis	Country of birth or linguistic and cultural background/religious background of participants	Study design	Relevant findings
Bairami et al. (2021)/Online	31.17/M—45, F—137/182	NR	Anglo/Caucasian (28), Asian (73), European (33), Middle Eastern (39), Other (9)/Muslims-Australians	Quantitative survey	In comparison with Anglo-Caucasian Muslim participants, CALD Muslim Australian participants were more likely to employ cultural attribution to mental illness. While Anglo participants more strongly endorsed mainstream professional treatment for mental illness over traditional and religious methods, Middle Eastern participants rated the effectiveness of Quran recitation significantly more than Anglo participants
Brijnath (2015)/Melbourne	Exp 38.3/M—13, F—15/28	Depression	Indian-Australian/Hindu, Muslim, Christian, Atheist or Agnostic, other	Qualitative	In comparison with Anglo Australian, Indian Australians drew meaning and peace from spirituality and religious activities. While Anglo-Australian participants made little mention of religion and spirituality, Indian-Australians reported using religious practices such as listening to religious songs and attending church/temple as mental health strategies. Religious faith was considered an important source of support and comfort for Indian Australian participants
	Cont 40.9/M—10, F—20/30	Depression	Anglo-Australian/Christian, Atheist or Agnostic, Other		
Chan (2009)/Sydney	Exp 18–79/M—110, F—146/256	Depression	Chinese Australian/NR	Mixed Method	Religious practices such as prayer were identified as a mental health strategy. Participants valued the support from the church and temples to support mental health. Religion and spirituality offered participants insight to change their perspective during adversity
	Cont 24–78/M—67, F—76/143	Depression	Australian/NR		
Du Plooy et al. (2019)/Online	46.2/M—634, F—695/1334	NR	Anglo (UK, USA, Canada, South Africa), Southern Asian (India, Pakistan, Bangladesh, Sri Lanka), Confucian Asian (China, Hong Kong, Singapore, Taiwan)/NR	Quantitative survey	Both the Southern Asian and Confucian Asian groups sought significantly more support from religious/church groups compared to the Anglo cultural group and having greater amounts of this support was linked to higher psychological flourishing. There were no significant associations between distress and support from various groups including church or religious groups
Fauk et al. (2022)/Locations in South Australia	18–60/M—10, F—10/20 Service providers—10	NR	The Democratic Republic of Congo (5) South Sudan (4) Liberia (3) Sierra Leone (3) Burundi (2) Ethiopia (1) Kenya (1) Somalia (1)/NR	Qualitative	Participants reported that African migrants' understanding of mental illness was influenced by their religious beliefs and not consistent with how mental illness is defined by Australian mental health services. Participants viewed this discrepancy as a barrier to accessing mental health services. Participants stressed involving local church and religious leaders in education and dissemination of information about mental illness. Since religious leaders are the first point of contact for African migrants, participants stressed educating them and involving them in the mental health care of these communities. It was also noted that African migrants would not feel shame to receive mental health information from religious leaders. Participants noted that the integration of some religious *values* (aspects) in mental health services may increase the acceptability
Hashemi et al. (2020)/Various locations in Queensland	30.41/M—199, F—183/382	NR	Middle Eastern/ Islam (348), Christian (5), Judaism (3), Others (6), No religion (20)	Quantitative, cross-sectional survey	Using structural equation modelling, this study explored associations between socioreligious predictors and the psychological well-being of migrants. Findings suggested that the religious identity of migrants was directly predictive of psychological well-being. Social connectedness with the ethnic community was a mediator in the association between religious identity and psychological well-being
Hocking (2021)/Melbourne	20–65/M—110, F—21/131	NR	Sri Lanka (49), Pakistan (36), Zimbabwe (21), Iraq (12), Afghanistan (10), Other (3)/NR	Qualitative	Participants identified religion as a protective factor and a buffer against hopelessness and supported mental health. Social support through the religious community helped participants to cope with stress and provided a sense of belonging. Religious practices such as prayers, rituals, reading scriptures and religious counselling were used as mental health strategies to manage stress. Adherence to a religious faith was a salient protective factor against suicide. Religious beliefs (for example, *their future was left to the will of Allah*) helped participants to adopt a positive outlook and supported mental health
Khawaja et al. (2008)/Brisbane	35/M—11, F—12/23	NR	Sudanese refugees/Christian (22), Islam (1)	Qualitative	Participants used their religion as a coping strategy to deal with the adversities and stress experienced throughout the migration process. Prayer was used as a mental health strategy and was used to combat stress. The sense of surrender through *placing their fate in God’s hands* helped in coping with stress and struggles related to migration. Religious community—church provided social, emotional, and material support. Religious beliefs helped participants to reappraise the adversities and contributed to coping with stress
Mitha and Adatia (2016)/NR	21.1/M—7, F—4/11 Community leaders—5	NR	NR/Australian Ismaili Muslim	Qualitative	Participants recognised drawing strength from religion through involvement in religious practices. They also found that religious community support was helpful for their mental health. Involving in religious activity provided a sense of belonging and helped in dealing with the feeling of isolation and sadness. Participants reported that the religious community provided a sense of comradery which helped with depression, anxiety, and loneliness. Involvement in religious activities such as *khidmat* (social service) helped to engage young participants and provided a meaningful role within the community. Religion was reported as a way of deriving meaning in life Religious practices such as *Dhikr* (chanting) and *bandagi* (meditation) and reading of scriptures were used as mental health strategies. Participants reported using religious practices as mental health strategies even outside formal religious places. Attending *jamatkhana* (religious place*)* was reported to be vital for the psychosocial well-being of participants and provided solace and comfort through spiritual/religious reflection and developing social support networks
Omar et al. (2017)/Melbourne	18–50/M—21, F—0/21	NR	Somalia (17), Ethiopia (1), Djibouti (3)/Muslim	Qualitative	Participants raised the lack of community religious support and connection by not having a *muezzin* (call to prayer). Religious practices such as reading/recitation of the Quran, and attending congregational prayers in a mosque were identified as *effective treatments* by participants. Participants reported religious beliefs as an important aspect to develop resilience to support mental health. Some participants suggested that the young generation who might have integrated both Australian and Somalian cultures were more likely to use both religious and mainstream mental health interventions
Prasad-Ildes and Ramirez (2006)/Brisbane	Age—NR/M—10, F—18/28	Major depression, schizophrenia, bipolar, anxiety and personality disorders	Egypt (2), Iraq (2) and Lebanon (2), Colombia (1), Guatemala (1), El Salvador (3), Ecuador (1), Spain (1), Chile (1) Bosnia (4), Filipinas (6) and Iran (4)/NR	Qualitative	A lack of understanding of consumers’ religious beliefs and practices among mental health professionals was identified. Participants reported the need for mental health literacy among religious readers. Participants noted that religious leaders can be a suitable medium for education and support regarding mental health. Training and education of religious leaders and facilitation of linkage between them and local mental health services were recommended as they are the first call for help for people with CALD backgrounds
Ridgway (2022)/Melbourne	26–43/M—0, F—9/9	NR	Albania (1), Indonesia (1), Hong Kong (1), India (2), Arica (1), America (2), UK (1)/Christian, Hindu, Buddhist, self-defined ‘fatalist’	Qualitative	Participating migrant women coped with the stress of divorce through their religiosity/spirituality. Communication and building/reinforcing the relationship with the divine through religious practices such as prayer helped them to *endure* adversity and *rebuild* their lives overseas. Religious beliefs helped to *reframe* their perspective on marital loss which helped in developing resilience. Through the lens of religion, participants were able to change the *narrative* of their loss and it provided hope for the continuation of life. Social and emotional support from the religious community and through collective chanting and group discussion of religious beliefs were reported to provide a *safe place* to the participants which supported their mental health
Said et al. (2021)/Melbourne	23.1/M—0, F—31/31	NR	Somali-Australian/NR	Qualitative	Participants reported a strong influence of religion (Islam) on their perception of mental illness. Some participants reported their faith as a *barrier* to seeking help. Mental illness was considered part of *God’s plan,* and an endurance test with the prospect of a *better afterlife*. The participants expressed that the older generation Somalis believed that God could cure mental illness through prayer and reading the Quran. Participants advocated seeking medical assistance in conjunction with religious and traditional therapies. The participants noted that clinicians should consider the belief (in both traditional remedies and western/medical treatment) of the Somali-Australian community when providing mental health care
Schweitzer et al. (2007)/Brisbane	29.77/M—9, F—4/13	NR	Sudanese refugees/Christian	Qualitative	Participants noted their religious beliefs as a source of strength and helped to cope with stress. Their belief in God provided *meaning* (perspective) in life which then helped to regain some control over their life. Participants reported using religious practices such as praying as a mental health strategy to help with the feeling of loneliness and sadness. A religious community such as a church also provided social, emotional, and material support
Stolk et al. (2015)/NR	Exp 44.84/M—10, F—9/19	Psychosis	Vietnamese-Australian/NR	Quantitative survey	The study compared the functioning of Vietnamese-Australian (low English proficiency) and Australian-born patients with psychosis. In the study sample more (84.2%) Vietnamese Australian participants rated spirituality or religion as important than Australian participants (53.3%). About half of Vietnamese Australian participants attributed mental illness to *supernatural causes*, none consulted traditional healers
	Cont 46.53/M—7, F—8/15	Psychosis	Australians (ethnicity—NR)/NR		
Youssef and Deane (2006)/Sydney	NR/M—19, F—16/35	NR	Arabic Australian-Egypt (18), Lebanese (16), Jordan (1)/Muslim, Christian	Qualitative	The participants demonstrated knowledge and perception of mental illness guided by religious beliefs. The participant reported that as per their religious law, a bridegroom can withdraw from a marriage contract if there is substantial proof of mental illness. This would affect the likelihood of marriage being proposed to any female member of that family since mental illness is considered a hereditary factor in the prospective bride’s family. Participants reported being more comfortable seeking help from religious leaders than mental health professionals. The role of religious leaders was emphasised as important and influential. Participants reported trust (confidentiality) and belief in the ‘spiritual healing power’ of religious leaders. Religious leaders were reported as the initial point of contact. Participants reported their beliefs on religious healing rituals such as reading verses from the Holy book

### Participant Characteristics

The review included 2711 participants from 16 studies, with 1414 (52%) being females. The cultural and linguistic backgrounds of participants were reported differently across all studies with the majority of studies reporting country of birth, while others reported the ethnicity of participants. In the included studies, most participants were of African origin (du Plooy et al., 2019; Fauk et al., 2022; Hocking, 2021; Khawaja et al., 2008; Omar et al., 2017; Said et al., 2021; Schweitzer et al., 2007) and from Middle Eastern background (Bairami et al., 2021; Hashemi et al., 2020; Hocking, 2021; Prasad-Ildes & Ramirez, 2006; Youssef & Deane, 2006). Of 16 included studies, nine studies reported the religious affiliation of participants (Bairami et al., 2021; Brijnath, 2015; Hashemi et al., 2020; Khawaja et al., 2008; Mitha & Adatia, 2016; Omar et al., 2017; Ridgway, 2022; Schweitzer et al., 2007; Youssef & Deane, 2006) with Islam being most commonly reported religion (Bairami et al., 2021; Brijnath, 2015; Hashemi et al., 2020; Khawaja et al., 2008; Mitha & Adatia, 2016; Omar et al., 2017; Youssef & Deane, 2006).

### Key Findings

The narrative synthesis of key findings from 16 included studies is summarised in three themes: the centralised role of religion/spirituality in supporting mental health; the use of religious/spiritual practices as mental health strategies; the role of religious leaders in the collaborative approach of mental health care.

#### The Centralised Role of Religion/Spirituality in Supporting Mental Health

Religion/spirituality appeared to play a central role in the mental well-being of people from CALD backgrounds. Religious identity was directly predictive of the psychological well-being of participants from Middle Eastern backgrounds in a cross-sectional quantitative survey (Hashemi et al., 2020). Religion/spirituality was reported to be important for Vietnamese Australian participants recovering from psychosis (Stolk et al., 2015) and was a source of comfort for Indian Australians with depression (Brijnath, 2015). Participants of included studies reported using their religion/spirituality as a coping strategy (Khawaja et al., 2008), a protective factor against depression and suicidal ideations (Hocking, 2021), and a source to develop mental resilience (Omar et al., 2017; Ridgway, 2022). Religion/spirituality provided *meaning* during adversities and distress (Brijnath, 2015; Mitha & Adatia, 2016; Schweitzer et al., 2007) and was reported as a source of strength against feelings of isolation and sadness (Mitha & Adatia, 2016).

Participants in some studies reported using their religious beliefs to alter their perspective on a stressful event, which improved their mental health (Chan, 2009; Hocking, 2021; Khawaja et al., 2008; Mitha & Adatia, 2016; Ridgway, 2022). For example, in a qualitative study with nine migrant women of various religious faiths, participants noted how their religious beliefs helped to *reframe* their thoughts about divorce, changed the narrative of their marital loss and provided hope for the continuation of life (Ridgway, 2022). Religious beliefs helped participants reappraise adversities and contributed to stress coping in another qualitative study with Sudanese refugees who were primarily of Christian faith (Khawaja et al., 2008).

Social support through the religious community was reported to be an important mediator of psychological well-being in several studies (Chan, 2009; du Plooy et al., 2019; Hocking, 2021; Khawaja et al., 2008; Mitha & Adatia, 2016; Omar et al., 2017; Ridgway, 2022; Schweitzer et al., 2007). Participants in some studies reported that the sense of camaraderie and belonging to a religious community helped with depression and anxiety (Mitha & Adatia, 2016) and provided them with a safe place (Mitha & Adatia, 2016; Ridgway, 2022) for spiritual/religious reflection and the development of social support networks.

Participants in various studies viewed mental illness through religious and cultural lenses (Bairami et al., 2021; Fauk et al., 2022; Said et al., 2021; Stolk et al., 2015; Youssef & Deane, 2006). In a quantitative survey of 182 participants, CALD Muslim Australian participants were more likely to use their religious and cultural beliefs to explain the aetiology of mental illness than non-CALD Anglo-Caucasian Muslim participants (Bairami et al., 2021). In a qualitative study, African migrants' understanding of mental illness was reported to be influenced by their religious beliefs and not consistent with how mental illness is defined by Australian mental health services (Fauk et al., 2022). This disparity in understanding of mental illness was identified as a barrier to accessing mental health services in two studies (Fauk et al., 2022; Said et al., 2021).

#### The Use of Religious/Spiritual Practices as Mental Health Strategies

In this review, participants in several studies identified religious/spiritual practices as mental health strategies (Bairami et al., 2021; Brijnath, 2015; Chan, 2009; Hocking, 2021; Khawaja et al., 2008; Mitha & Adatia, 2016; Omar et al., 2017; Ridgway, 2022; Said et al., 2021; Schweitzer et al., 2007; Youssef & Deane, 2006). CALD Muslim Australian participants rated the effectiveness of religious practices such as Quran recitation significantly higher than non-CALD Anglo/Caucasian Muslim participants in a quantitative survey (Bairami et al., 2021). Similarly, while Anglo Australian participants made little mention of religion and spirituality in another qualitative survey, Indian Australians reported using religious practices such as listening to religious songs and attending church/temple as mental health strategies (Brijnath, 2015). Religious/spiritual practices such as reading scriptures, prayers, chanting, (*Dhikr*—Mitha & Adatia, 2016) and meditation (*Bandagi*—Mitha & Adatia, 2016) were reported as effective mental health strategies in the majority of studies (Bairami et al., 2021; Brijnath, 2015; Chan, 2009; Hocking, 2021; Khawaja et al., 2008; Mitha & Adatia, 2016; Omar et al., 2017; Ridgway, 2022; Said et al., 2021; Schweitzer et al., 2007; Youssef & Deane, 2006). The participants of one study reported using religious practices as mental health strategies even outside of religious settings (Mitha & Adatia, 2016).

#### The Role of Religious Leaders in the Collaborative Approach of Mental Health Care

The role of religious leaders in mental health care was emphasised in several studies (Fauk et al., 2022; Prasad-Ildes & Ramirez, 2006; Youssef & Deane, 2006). In a study with African migrants and service providers, participants emphasised the importance of involving local church and religious leaders in the education and dissemination of information about mental illness (Fauk et al., 2022). Participants in a few studies (Fauk et al., 2022; Youssef & Deane, 2006) highlighted the need for religious leaders' education and training because they were the first point of contact for some CALD communities.

The importance of involving religious leaders in the mental health care of the CALD population was also highlighted by the participants in several studies (Fauk et al., 2022; Prasad-Ildes & Ramirez, 2006; Youssef & Deane, 2006). According to one study, African migrants were reported to be open to receiving mental health care and information from religious leaders (Fauk et al., 2022). Similarly, in another study with Arabic Australians, participants reported being more comfortable in seeking help from religious leaders than mental health professionals and the role of religious leaders was emphasised as important and influential (Youssef & Deane, 2006).

Participants in some studies emphasised the significance of incorporating religious/spiritual components into mainstream mental health interventions, as well as the involvement of religious leaders in the collaborative approach to mental health care (Fauk et al., 2022; Prasad-Ildes & Ramirez, 2006; Said et al., 2021). A lack of understanding of consumers’ religious beliefs and practices among mental health professionals was identified in one study (Prasad-Ildes & Ramirez).

## Discussion

This study aimed to critically review the current evidence regarding the need for the inclusion of religion and spirituality in the mental health care for the CALD population in Australia. The findings of the review highlighted the integral role of religion and spirituality in the mental well-being of CALD communities. Evidence from the included studies suggested that religion/spirituality was related to better mental health resilience and coping among people from CALD backgrounds. Consistent with this finding, in several other Australian studies, participants from CALD backgrounds used religion/spirituality to cope with stress related to physical illness (Ahmad et al., 2022; Kirby et al., 2018; Sellappah et al., 2001) and caregiving (Benedetti et al., 2013). Though not included in the review (due to the age range of the participants), in another Australian qualitative study, young people from CALD backgrounds noted religion/spirituality as a source of strength and resilience in dealing with mental health issues (Gorman et al., 2003).

There were several ways people from CALD backgrounds utilised religion/spirituality to support their mental health. Religious/spiritual beliefs appeared to provide them with an alternative perspective on stressful situations, which might have contributed to their mental well-being. Participants in some of the included studies used religious beliefs to reframe their stressful thoughts through the lens of their religious beliefs, which then supported mental health (Chan, 2009; Hocking, 2021; Khawaja et al., 2008; Mitha & Adatia, 2016; Ridgway, 2022). Supporting mental health through changing unhelpful thought patterns is the basic strategy of the most commonly used psychological intervention (DeRubeis et al., 1990). Since religious beliefs were reported to help change the outlook, faith-adapted psychological interventions (Abdul-Hamid & Hughes, 2015; Anderson et al., 2015), that incorporate religious beliefs into conventional psychological therapies may be an acceptable choice of intervention for CALD communities.

Although religion/spirituality was noted to be largely beneficial for the mental health of the CALD population, participants in some studies reported viewing mental illness from their religious perspective, which potentially posed a barrier to accessing mental health care. For example, participants primarily from African and Middle Eastern backgrounds and identified as Muslims, perceived mental illness through the lens of their religious and cultural beliefs (Bairami et al., 2021; Fauk et al., 2022; Said et al., 2021; Youssef & Deane, 2006) and preferred religious interventions over mainstream mental health care (Bairami et al., 2021). Consistent with this finding, in a previous review, mental health literacy issues were prominent in Muslim communities irrespective of their country of origin and despite their formal education (Gurr & Meiser, 1996).

While religious beliefs about mental illness were reported as a barrier to accessing mental health services in one study (Said et al., 2021) of this review, participants from CALD communities were open to using religious practices as mental health strategies. Of 16 included studies in the review, participants from 11 studies (Bairami et al., 2021; Brijnath, 2015; Chan, 2009; Hocking, 2021; Khawaja et al., 2008; Mitha & Adatia, 2016; Omar et al., 2017; Ridgway, 2022; Said et al., 2021; Schweitzer et al., 2007; Youssef & Deane, 2006) were either already using religious practices to support mental health or endorsed them as viable mental health strategies. This aligns with the finding of a study with young Somalians, who were reluctant to use mental health services but were willing to receive religious interventions (Johnsdotter et al., 2011). While mental health education and awareness remain important, incorporating well-evidenced religious/spiritual intervention into mental health care may increase service uptake among CALD communities.

The language barrier is another reason why people with CALD backgrounds are less likely to access healthcare services (Au et al., 2019; Henderson et al., 2011; Minas et al., 2013; Samuel et al., 2018). The body-based mental health interventions, which require little to no language skills, can be used as a supplement or alternative interventions for CALD populations with limited language skills. Some of the body-based religious/spiritual practices have substantial empirical evidence of mental health benefits, such as yoga, chanting, and breathwork (e.g. Brinsley et al., 2020; Lynch et al., 2018; Malviya et al., 2022a). As noted earlier, participants of several studies in this review emphasised the need for integrating religious/spiritual interventions into mainstream mental health care (Fauk et al., 2022; Prasad-Ildes & Ramirez, 2006; Said et al., 2021). Given the language barrier and preference for religious interventions, body focussed religious/spiritual practice (also termed sensorimotor religious/spiritual practice (Malviya et al., 2022b) can be a viable alternative for some CALD communities.

Sensorimotor religious/spiritual practice such as recitation of scriptures is well evidenced to support mental health (Babamohamadi et al., 2015; Mahmood et al., 2007; Rafique et al., 2019) of people from the Islamic background. As such, sensorimotor religious/spiritual practices such as recitation may be used as an alternative and/or supportive intervention for Arab Australians of Islamic faith who are hesitant to use counselling and other conversational therapies (Al-Issa et al., 2000; Omar et al., 2017). Additionally, three sensorimotor religious/spiritual practices (yoga, chanting, and breathwork) were identified as potentially viable interventions to be used in clinical services in a recent survey of Australian mental health professionals (Malviya et al., 2022c). Given the reported preference for religious/spiritual interventions and reluctance to use conventional interventions among people from CALD backgrounds, sensorimotor religious/spiritual practices may be integrated into mainstream mental health care for CALD populations.

Participants of several included studies in the review emphasised the importance of involving religious leaders in the mental health care (Fauk et al., 2022; Prasad-Ildes & Ramirez, 2006; Youssef & Deane, 2006) for CALD communities. Possibly due to religious beliefs associated with mental illness, participants primarily from African and Middle Eastern backgrounds felt more comfortable receiving care from religious leaders than mental health professionals (Fauk et al., 2022; Youssef & Deane, 2006). Training and education of religious leaders, as recommended by some scholars (e.g. Koenig, 2008), and linking them with mental health services to provide collaborative care may benefit people of CALD background.

Participants in one study of this review identified the need for rigorous and in-depth training for mental health professionals regarding different religions (Prasad-Ildes & Ramirez, 2006). Though participants of this study were people with mental health concerns (Prasad-Ildes & Ramirez, 2006), a similar requirement of nuanced and in-depth training regarding diverse religions and cultures was also identified in a qualitative survey with Australian health workers (Lindegaard Moensted & Day, 2022). Since one of the reasons CALD people may not access mental health care is that health professionals do not understand their religious and cultural beliefs (Mitchell et al., 1998; Tobin, 2000), training and education of health professionals are essential.

## Recommendations for Clinical Practice

The following recommendations can be drawn from the rapid review. Exploration of religious/spiritual orientation is noted to be critical for CALD community mental health assessment and care. Faith-adapted psychological interventions may be an alternative for CALD people willing to receive psychological therapies. Sensorimotor religious/spiritual practices (e.g. breathwork, chanting/recitation, and yoga) may be offered as a supportive and/or alternative intervention to the CALD people. Religious leaders' education and training, as well as their involvement in the provision of mental health care for the CALD population, particularly those from Africa and the Middle East, may be considered. Mental health professionals who provide care to the CALD population may be required to receive in-depth training on various religions and cultures.

## Limitations

The review considered the CALD population including voluntary migrants, refugees, and other humanitarian entrants. It is important to note that there is diversity at many levels within the CALD group. For example, mental health care for a refugee displaced from a war-affected country would differ from that of a voluntary migrant who came through skilled migration. Also, people from different religious and/or ethnic backgrounds may have unique mental health needs. As a result, while the review's findings may be relevant to some extent for the overall CALD population, they should only be regarded as indicative. More focussed research is needed to identify the religious/spiritual needs of mental health care for specific groups within CALD communities. Only studies on religion and spirituality were included in this review. Because there was no clear distinction in the literature between cultural and religious aspects, some relevant research that reported religious or spiritual components as cultural traits may have been overlooked.

## Conclusion

This rapid review provides some insight into the importance of the inclusion of religious/spiritual aspects in the mental health care of CALD communities in Australia. Available evidence suggests that people from CALD backgrounds draw strength and comfort from their religion to support mental health. Religious/spiritual practices were identified as effective mental health strategies by CALD people. Religious leaders may play an important role in mental health education and care for CALD communities. Involvement of religious leaders in mental health services, and integration of religious/spiritual practices in mainstream mental health interventions may improve mental health care for people with CALD backgrounds. Mental health professionals who provide care to CALD communities, should receive training and education about various religions and cultures.

## Supplementary Information

Below is the link to the electronic supplementary material.

Supplementary file1 (DOCX 24 KB)

## References

[CR1] Abbott A, Silles M (2016). Determinants of international student migration. The World Economy.

[CR2] Abdul-Hamid WK, Hughes JH (2015). Integration of religion and spirituality into trauma psychotherapy: An example in Sufism?. Journal of EMDR Practice and Research.

[CR3] Ahmad A, Khan MU, Aslani P (2022). The role of religion, spirituality and fasting in coping with diabetes among Indian migrants in Australia: A qualitative exploratory study. Journal of Religion & Health.

[CR4] Al-Issa I, Al Zubaidi A, Bakal D, Fung TS (2000). Beck Anxiety Inventory symptoms in Arab college students. Arab Journal of Psychiatry.

[CR5] Anderson N, Heywood-Everett S, Siddiqi N, Wright J, Meredith J, McMillan D (2015). Faith-adapted psychological therapies for depression and anxiety: Systematic review and meta-analysis. Journal of Affective Disorders.

[CR6] Au M, Anandakumar AD, Preston R, Ray RA, Davis M (2019). A model explaining refugee experiences of the Australian healthcare system: A systematic review of refugee perceptions. BMC International Health and Human Rights.

[CR8] Australian Bureau of Statistics. (2022a). *Cultural diversity:Census*. Retrived from, https://www.abs.gov.au/statistics/people/people-and-communities/cultural-diversity-census/2021

[CR9] Australian Bureau of Statistics. (2022b). *Proficiency in spoken English*. Retrived from, https://www.abs.gov.au/census/guide-census-data/census-dictionary/2021/variables-topic/cultural-diversity/proficiency-spoken-english-englp

[CR7] Australian Bureau of Statistics. (2022c). *Religious affiliation in Australia*. Australian Government. Retrived from, https://www.abs.gov.au/articles/religious-affiliation-australia

[CR10] Babamohamadi H, Sotodehasl N, Koenig HG, Jahani C, Ghorbani R (2015). The effect of Holy Qur’an recitation on anxiety in hemodialysis patients: A randomized clinical trial. Journal of Religion and Health.

[CR11] Bairami K, Spivak BL, Burke LM, Shepherd SM (2021). Exploring mental illness attributions and treatment-seeking beliefs in a diverse Muslim-Australian sample. Clinical Psychologist.

[CR12] Beaini D, Shepherd SM (2022). Working with Arab women with PTSD: What do we know?. Australian Psychologist.

[CR13] Benedetti R, Cohen L, Taylor M (2013). “There's really no other option”: Italian Australians’ experiences of caring for a family member with dementia. Journal of Women & Aging.

[CR14] Bhugra D (2004). Migration and mental health. Acta Psychiatrica Scandinavica.

[CR15] Brijnath B (2015). Applying the CHIME recovery framework in two culturally diverse Australian communities: Qualitative results. International Journal of Social Psychiatry.

[CR16] Brinsley J, Schuch F, Lederman O, Girard D, Smout M, Immink MA, Stubbs B, Firth J, Davison K, Rosenbaum S (2020). Effects of yoga on depressive symptoms in people with mental disorders: A systematic review and meta-analysis. British Journal of Sports Medicine.

[CR17] Carolan M, Cassar L (2010). Antenatal care perceptions of pregnant African women attending maternity services in Melbourne, Australia. Midwifery.

[CR18] Chan B (2009). Capitalising on the social resources within culturally and linguistically diverse communities for mental health promotion: Stories of Australian Chinese people. Australian Journal of Primary Health.

[CR19] Department of Health and Aged Care. (2018). *Mental health services for people of culturally and linguistically diverse (CALD) backgrounds*. Department of Health and Aged Care: Australian Government. Retrived from, https://www.health.gov.au/resources/publications/mental-health-services-for-people-of-culturally-and-linguistically-diverse-cald-backgrounds

[CR20] DeRubeis RJ, Evans MD, Hollon SD, Garvey MJ, Grove WM, Tuason VB (1990). How does cognitive therapy work? Cognitive change and symptom change in cognitive therapy and pharmacotherapy for depression. Journal of Consulting and Clinical Psychology.

[CR21] du Plooy DR, Lyons A, Kashima ES (2019). The effect of social support on psychological flourishing and distress among migrants in Australia. Journal of Immigrant & Minority Health.

[CR22] Fauk NK, Ziersch A, Gesesew H, Ward PR, Mwanri L (2022). Strategies to improve access to mental health services: Perspectives of African migrants and service providers in South Australia. SSM - Mental Health.

[CR23] Gorman D, Brough M, Ramirez E (2003). How young people from culturally and linguistically diverse backgrounds experience mental health: Some insights for mental health nurses. International Journal of Mental Health Nursing.

[CR24] Gurr R, Meiser B (1996). Non-English-speaking persons’ perceptions of mental illness and associated information needs: an exploratory study. Health Promotion Journal of Australia.

[CR25] Hashemi N, Marzban M, Sebar B, Harris N (2020). Religious identity and psychological well-being among middle-eastern migrants in Australia: The mediating role of perceived social support, social connectedness, and perceived discrimination. Psychology of Religion and Spirituality.

[CR26] Henderson S, Kendall E, See L (2011). The effectiveness of culturally appropriate interventions to manage or prevent chronic disease in culturally and linguistically diverse communities: A systematic literature review. Health Social Care Community.

[CR27] Hocking DC (2021). To strive, to seek, to find, and not to yield: Narratives on the road to asylum. Transcultural Psychiatry.

[CR28] Hong QN, Fàbregues S, Bartlett G, Boardman F, Cargo M, Dagenais P, Gagnon M-P, Griffiths F, Nicolau B, O’Cathain A, Rousseau M-C, Vedel I, Pluye P (2018). The Mixed Methods Appraisal Tool (MMAT) version 2018 for information professionals and researchers. Education for Information.

[CR29] Johnsdotter S, Ingvarsdotter K, Östman M, Carlbom A (2011). Koran reading and negotiation with jinn: Strategies to deal with mental ill health among Swedish Somalis. Mental Health, Religion & Culture.

[CR30] Khangura S, Konnyu K, Cushman R, Grimshaw J, Moher D (2012). Evidence summaries: The evolution of a rapid review approach. Systematic Reviews.

[CR31] Khatri RB, Assefa Y (2022). Access to health services among culturally and linguistically diverse populations in the Australian universal health care system: Issues and challenges. BMC Public Health.

[CR32] Khawaja NG (2007). An investigation of the psychological distress of Muslim migrants in Australia. Journal of Muslim Mental Health.

[CR33] Khawaja NG, White KM, Schweitzer R, Greenslade J (2008). Difficulties and coping strategies of Sudanese refugees: A qualitative approach. Transcultural Psychiatry.

[CR34] Kirby E, Lwin Z, Kenny K, Broom A, Birman H, Good P (2018). "It doesn't exist...": Negotiating palliative care from a culturally and linguistically diverse patient and caregiver perspective. BMC Palliative Care.

[CR35] Koenig HG (2008). Religion and mental health: What should psychiatrists do?. Psychiatric Bulletin.

[CR36] Lindegaard Moensted M, Day C (2022). Operationalising cultural competency in the context of substance use treatment: A qualitative analysis. Drugs: Education, Prevention & Policy.

[CR37] Lynch J, Prihodova L, Dunne PJ, Carroll Á, Walsh C, McMahon G, White B (2018). Mantra meditation for mental health in the general population: A systematic review. European Journal of Integrative Medicine.

[CR38] Mahmood N, Malook N, Riaz A (2007). The effect of rhythmic Quranic recitation on depression. Journal of Behavioural Sciences.

[CR39] Malviya, S. (2022, November 22). Need for integration of religion and spirituality in mental health care of culturally and linguistically diverse population in Australia: A rapid review. Open Science Framework. Retrieved from osf.io/2wuqh. 10.1007/s10943-023-01761-3 PMC10366032 36780111

[CR40] Malviya S, Meredith P, Zupan B, Kerley L (2022). Identifying alternative mental health interventions: a systematic review of randomized controlled trials of chanting and breathwork. Journal of Spirituality in Mental Health.

[CR41] Malviya S, Zupan B, Meredith P (2022). Evidence of religious/spiritual singing and movement in mental health: A systematic review. Complementary Therapies in Clinical Practice.

[CR42] Malviya S, Zupan B, Meredith P (2022). Alternative interventions in clinical mental health settings: A survey of mental health professionals' perceptions. Complementary Therapies in Clinical Practice.

[CR43] Minas H, Kakuma R, Too LS, Vayani H, Orapeleng S, Prasad-Ildes R, Turner G, Procter N, Oehm D (2013). Mental health research and evaluation in multicultural Australia: Developing a culture of inclusion. International Journal of Mental Health Systems.

[CR44] Mitchell P, Malak A, Small D (1998). Bilingual professionals in community mental health services. Australian and New Zealand Journal of Psychiatry.

[CR45] Mitha K, Adatia S (2016). The faith community and mental health resilience amongst Australian Ismaili Muslim youth. Mental Health, Religion & Culture.

[CR46] Omar YS, Kuay J, Tuncer C (2017). ‘Putting your feet in gloves designed for hands’: Horn of Africa Muslim men perspectives in emotional wellbeing and access to mental health services in Australia. International Journal of Culture & Mental Health.

[CR47] Page MJ, McKenzie JE, Bossuyt PM, Boutron I, Hoffmann TC, Mulrow CD, Shamseer L, Tetzlaff JM, Akl EA, Brennan SE (2021). The PRISMA 2020 statement: An updated guideline for reporting systematic reviews. Systematic Reviews.

[CR48] Popay, J., Roberts, H., Sowden, A., Petticrew, M., Arai, L., Rodgers, M., Britten, N., Roen, K., & Duffy, S. (2006). *Guidance on the conduct of narrative synthesis in systematic reviews: A product from the Economic and Social Research Council (ESRC) methods programme*. United Kingdom. 10.13140/2.1.1018.4643

[CR49] Prasad-Ildes R, Ramirez E (2006). What CALD consumers say about mental illness prevention. Australian e-Journal for the Advancement of Mental Health.

[CR50] Rafique R, Anjum A, Raheem SS (2019). Efficacy of Surah Al-Rehman in managing depression in Muslim women. Journal of Religion and Health.

[CR51] Ridgway A (2022). “I had faith”: Migrant women’s use of religion and spirituality to reframe their divorce and for self-reinvention. Journal of Divorce & Remarriage.

[CR52] Said M, Boardman G, Kidd S (2021). Barriers to accessing mental health services in Somali-Australian women: A qualitative study. International Journal of Mental Health Nursing.

[CR53] Samuel S, Advocat J, Russell G (2018). Health seeking narratives of unwell Sri Lankan Tamil refugees in Melbourne Australia. Australian Journal of Primary Health.

[CR54] Schweitzer R, Greenslade J, Kagee A (2007). Coping and resilience in refugees from the Sudan: A narrative account. Australian & New Zealand Journal of Psychiatry.

[CR55] Sellappah S, Kristjanson LJ, Maltby H (2001). Cancer in Western Australian Indian families: concerns and coping strategies. International Journal of Palliative Nursing.

[CR56] Stolk Y, Sevar K, Tran N, Mancuso SG, Chopra P, Castle D (2015). A comparative study of the economic and social functioning of Vietnamese-Australians with low English proficiency living with psychotic illness. International Journal of Social Psychiatry.

[CR57] Tobin M (2000). Developing mental health rehabilitation services in a culturally appropriate context. Australian Health Review.

[CR58] Tricco, A.C., Langlois, E.V., & Straus, S.E. (Eds.). (2017). *Rapid reviews to strengthen health policy and systems: A practical guide.* World Health Organisation. Retrieved from http://apps.who.int/iris/bitstream/handle/10665/258698/9789241512763-eng.pdf?sequence=1

[CR59] Youssef J, Deane FP (2006). Factors influencing mental-health help-seeking in Arabic-speaking communities in Sydney, Australia. Mental Health, Religion & Culture.

