# Supplementary material for: The Need for Integration of Religion and Spirituality into the Mental Health Care of Culturally and Linguistically Diverse Populations in Australia: A Rapid Review
Source: J Relig Health. 2023 Feb 13;62(4):2272–96. doi: 10.1007/s10943-023-01761-3 (PMC10366032; doi:10.1007/s10943-023-01761-3)
Supplement: Supplementary file 1 — Supplementary file1 (DOCX 24 KB) [file 10943_2023_1761_MOESM1_ESM.docx]

**Need for integration of religion and spirituality into the mental health care of culturally and linguistically diverse populations in Australia: A rapid review**

**Appendix 1**

**Search strategy for PsycINFO**

| **#** | **Query** | **Results from 10 Aug 2022** |
| --- | --- | --- |
| 1 | exp Mental Health/ | 81,727 |
| 2 | (Mental disorders or Mental health program or Mental health care or Mental health assessment or Community Mental Health Services).mp. | 212,168 |
| 3 | (Psychotic Disorders or Mental disorders or screening mental disorder).mp. | 210,218 |
| 4 | exp Major Depression/ or exp Well Being/ or exp Stress/ or exp Mental Health/ | 384,187 |
| 5 | 1 or 2 or 3 or 4 | 574,891 |
| 6 | Immigrants.mp. or exp Immigration/ | 31,756 |
| 7 | [refugees.mp](http://refugees.mp/). or exp Refugees/ | 10,311 |
| 8 | exp Diversity/ or exp Minority Groups/ or exp Multiculturalism/ or exp Cultural Diversity/ | 40,419 |
| 9 | exp Cultural Diversity/ | 2,344 |
| 10 | exp Linguistics/ | 114,764 |
| 11 | CALD.mp. [mp=title, abstract, heading word, table of contents, key concepts, original title, tests & measures, mesh word] | 195 |
| 12 | non-English [speaking.mp](http://speaking.mp/). [mp=title, abstract, heading word, table of contents, key concepts, original title, tests & measures, mesh word] | 1,025 |
| 13 | [migrant.mp](http://migrant.mp/). [mp=title, abstract, heading word, table of contents, key concepts, original title, tests & measures, mesh word] | 7,655 |
| 14 | [refugee.mp](http://refugee.mp/). [mp=title, abstract, heading word, table of contents, key concepts, original title, tests & measures, mesh word] | 6,715 |
| 15 | [ethnic.mp](http://ethnic.mp/). [mp=title, abstract, heading word, table of contents, key concepts, original title, tests & measures, mesh word] | 117,691 |
| 16 | "Chinese Australian".mp. [mp=title, abstract, heading word, table of contents, key concepts, original title, tests & measures, mesh word] | 66 |
| 17 | "Indian Australian".mp. [mp=title, abstract, heading word, table of contents, key concepts, original title, tests & measures, mesh word] | 7 |
| 18 | "Arabic Australian".mp. [mp=title, abstract, heading word, table of contents, key concepts, original title, tests & measures, mesh word] | 3 |
| 19 | "African Australian".mp. [mp=title, abstract, heading word, table of contents, key concepts, original title, tests & measures, mesh word] | 12 |
| 20 | "culturally diverse".mp. [mp=title, abstract, heading word, table of contents, key concepts, original title, tests & measures, mesh word] | 4,061 |
| 21 | "linguistically diverse".mp. [mp=title, abstract, heading word, table of contents, key concepts, original title, tests & measures, mesh word] | 1,964 |
| 22 | Indian.mp. [mp=title, abstract, heading word, table of contents, key concepts, original title, tests & measures, mesh word] | 20,603 |
| 23 | [multicultural.mp](http://multicultural.mp/). [mp=title, abstract, heading word, table of contents, key concepts, original title, tests & measures, mesh word] | 15,946 |
| 24 | Chinese.mp. [mp=title, abstract, heading word, table of contents, key concepts, original title, tests & measures, mesh word] | 62,900 |
| 25 | Malaysian.mp. [mp=title, abstract, heading word, table of contents, key concepts, original title, tests & measures, mesh word] | 2,086 |
| 26 | Vietnamese.mp. [mp=title, abstract, heading word, table of contents, key concepts, original title, tests & measures, mesh word] | 2,995 |
| 27 | Arab.mp. [mp=title, abstract, heading word, table of contents, key concepts, original title, tests & measures, mesh word] | 6,437 |
| 28 | Iraqi.mp. [mp=title, abstract, heading word, table of contents, key concepts, original title, tests & measures, mesh word] | 1,876 |
| 29 | Irani.mp. [mp=title, abstract, heading word, table of contents, key concepts, original title, tests & measures, mesh word] | 27 |
| 30 | Syrian.mp. [mp=title, abstract, heading word, table of contents, key concepts, original title, tests & measures, mesh word] | 1,595 |
| 31 | Afghan.mp. [mp=title, abstract, heading word, table of contents, key concepts, original title, tests & measures, mesh word] | 1,458 |
| 32 | "Middle Eastern".mp. [mp=title, abstract, heading word, table of contents, key concepts, original title, tests & measures, mesh word] | 1,417 |
| 33 | Sudanese.mp. [mp=title, abstract, heading word, table of contents, key concepts, original title, tests & measures, mesh word] | 388 |
| 34 | Jordanian.mp. [mp=title, abstract, heading word, table of contents, key concepts, original title, tests & measures, mesh word] | 1,213 |
| 35 | Somalian.mp. [mp=title, abstract, heading word, table of contents, key concepts, original title, tests & measures, mesh word] | 29 |
| 36 | 6 or 7 or 8 or 9 or 11 or 12 or 13 or 14 or 15 or 16 or 17 or 18 or 19 or 20 or 21 or 22 or 23 or 24 or 25 or 26 or 27 or 28 or 29 or 30 or 31 or 32 or 33 or 34 or 35 | 273,329 |
| 37 | exp Religion/ | 78,797 |
| 38 | Spirituality.mp. or exp Spirituality/ | 27,867 |
| 39 | Religion.mp. or (exp "Rituals (Religion)"/ or exp Religion/ or exp "Confession (Religion)"/ or exp Religious Beliefs/ or exp Religious Groups/) [mp=title, abstract, heading word, table of contents, key concepts, original title, tests & measures, mesh word] | 97,856 |
| 40 | 37 or 38 or 39 | 111,556 |
| 41 | Australia.mp. | 44,878 |
| 42 | Australian.mp. | 30,700 |
| 43 | Australians.mp. | 3,178 |
| 44 | 41 or 42 or 43 | 60,471 |
| 45 | 5 and 36 and 40 and 44 | 40 |

**Appendix 2**

**Quality assessment of included studies**

| **Mixed Method Studies** | | | | | | |
| --- | --- | --- | --- | --- | --- | --- |
| **Study** | **Total Score** | **Is there an adequate rationale for using a mixed methods design to address the research question?** | **Are the different components of the study effectively integrated to answer the research question?** | **Are the outcomes of the integration of qualitative and quantitative components adequately interpreted?** | **Are the divergences and inconsistencies between quantitative and qualitative results adequately addressed?** | **Do the different components of the study adhere to the quality criteria of each tradition of the methods involved** |
| Chan, 2009 | 5/5 | Yes | Yes | Yes | Yes | Yes |
| **Qualitative Study** | | | | | | |
| **Study** | **Total Score** | **Is the qualitative approach appropriate to answer the research question?** | **Are the qualitative data collection methods adequate to address the research question?** | **Are the findings adequately derived from the data?** | **Is the interpretation of results sufficiently substantiated by data?** | **Do the different components of the study adhere to the quality criteria of each tradition of the methods involved** |
| Brijnath, 2015 | 4/5 | Yes | Yes | Yes | Yes | Cannot tell |
| Fauk et al., 2022 | 5/5 | Yes | Yes | Yes | Yes | Yes |
| Hocking D.C., 2021 | 5/5 | Yes | Yes | Yes | Yes | Yes |
| Khawaja et al., 2008 | 4/5 | Yes | Yes | Yes | Yes | Cannot tell |
| Mitha & Adalia, 2016 | 3/5 | Yes | No | Yes | Yes | Cannot tell |
| Omar et al., 2017 | 4/5 | Yes | No | Yes | Yes | Yes |
| Prasad-Ildes & Ramirez, 2006 | 2/5 | Yes | Yes | No | No | No |
| Ridgway A. 2022 | 3/5 | Yes | Yes | Yes | No | No |
| Said et al., 2021 | 3/5 | Yes | Yes | Yes | No | No |
| Schweitzer et al., 2007 | 4/5 | Yes | Yes | Yes | Yes | No |
| Youssef & Deane, 2006 | 3/5 | Yes | Yes | Yes | No | No |
| **Quantitative Studies** | | | | | | |
| **Study** | **Total Score** | **Is the sampling strategy relevant to address the research question?** | **Is the sample representative of the target population?** | **Are the measurements appropriate?** | **Is the risk of nonresponse bias low?** | **Is the statistical analysis appropriate to answer the research question?** |
| Bairami et al., 2021 | 4/5 | Yes | Yes | Yes | Cannot tell | Yes |
| Du Plooy et al., 2019 | 4/5 | Yes | Yes | Yes | No | Yes |
| Hashemi et al., 2020 | 4/5 | Yes | Yes | Yes | Cannot tell | Yes |
| Stolk et al., 2014 | 5/5 | Yes | Yes | Yes | Yes | Yes |

Note: Yes-1; No-0; Cannot tell-0
